# Quantum agriculture and experimental detection of wheat flour quality using thermal image technology

**DOI:** 10.1016/j.heliyon.2023.e19899

**Published:** 2023-09-06

**Authors:** Rosa Bernardini Papalia, Daniele Gullà, Enzo Nastati

**Affiliations:** a Department of Statistical Sciences, University of Bologna, Italy; b INBB – Biostructures and Biosystems National Institute, Roma, Italy; c Arc of Life, Ariano Nel Polesine, Ro, Italy

**Keywords:** Quantum agriculture, Crop productivity, Functional information analysis

## Abstract

Crop quality and productivity are the fundamental strategies of agricultural practice and technology; consequently, one of the priorities in agriculture is to be aware of new and innovative field experiments and production practices. The effectiveness of innovative practices and resources is influenced by a multitude of factors. This study aimed to propose a new quantum-based approach in agriculture using homoeopathic methodology which incorporates theories and concepts of quantum physics and employs a wave-based methodology for the functional measurement phase. A novel utilization of thermal imaging technology is suggested, wherein each pixel of the image indicates the wave parameters, frequency, or amplitude of wave, is proposed to analyse the functional information of the plant. The relationship between the new quantum-agriculture-based method and the functional characteristics of flour as measured by bio-photonic emissions was estimated, and the findings of this preliminary study on wheat flour are presented. Our preliminary results (i) confirm the superior performance of quantum agriculture (QA) based proposed soil and plant treatments, and (ii) suggest that quantitative analysis based on precise measurements of biophoton emission will provide a novel reliable tool for monitoring the quality of wheat in the future. Further investigations are required to replicate the results of this study under different environmental conditions. Additionally, incorporating comparative chemical analyses that would enhance our knowledge of the proposed agronomic practice.

## Highlights

• Quantum agriculture (QA) treatments demonstrate superior performance.
• Relationship between biophoton emissions and wheat quality assessed.
• QA improved the gluten index of the test variety of wheat.
• Quantum treated flour samples presented greater vitality.

## 1 Introduction

Agriculture encompasses several methodologies, including conventional, integrated, organic, biodynamic, and homoeopathic. Conventional agriculture uses chemical-based herbicides and pesticides to control plant diseases and fertilisers to ensure the availability of nutrients in soil, resulting in environmental and groundwater pollution and pesticide residues in the food chain.

Organic farming is a sustainable form of agriculture that does not use synthetic pesticides and fertilisers; instead, agronomic practices and organic products are employed for weed control and fertilisation, respectively.

Biodynamic agriculture is a subset of organic farming but has superior characteristics due to the use of cosmic forces captured and fixed by horn manure and horn silica, which are prepared on the farm, beginning with the composting and humification of manure generally produced on the same farm. In fact, the biodynamic farm incorporates Dual, which is prepared from cattle manure by adding the appropriate biodynamic preparations forming a self-sustaining closed loop where the farms’ own resources are used to produce fertiliser. The treatment of plants adheres to homoeopathic principles; in fact, biodynamic farms employ the dynamisation process of biodynamic preparations. From the perspective of biodynamic agriculture, nitrogen conveys vital information to plants. The different forms of nitrogen and their significance in agriculture are well known. The nitrogen that is fixed from air into the soil by legumes is fundamental for both the enrichment of soils and nutrition of other plants. This naturally available nitrogen has intrinsic characteristics completely different from those of chemical-mineral fertilisation that the plant is “forced” to absorb via the circulating solution.

It is assumed that uptake of increasing doses of chemically synthesised nitrogen by wheat leads to the formation of proteins “with different degrees of harmonisation” within the same seed, thereby inducing gluten modification (especially of less “structured” gliadins), which is consequently intolerant for the individuals whose system recognises these alterations and modifications.

Homoeopathic agriculture [1] uses the same homoeopathic products that are recommended for humans, appropriately diluted according to the method proposed by Hahnemann, with excellent results not only in terms of improved plant health, but also enhanced yield per hectare to the highest level [2].

More recently, in the United States, a new experimental approach known as quantum agriculture (QA), has been used with great success, which is the subject of this study.

To date, both organic and industrial agriculture have been based on particulate matter approaches from chemistry and biology. This approach focuses on the nature of the individual components of physical systems, such as atoms, plant genes, soil-borne diseases, or water pollutants. In contrast, QA incorporates the theories and concepts of quantum physics and biology, which adopt an approach based on waves and information carried by biophotons.

In recent years, there has been an unprecedented increase in the knowledge and comprehension of quantum theory. Quantum theory (also known as quantum physics or quantum mechanics) is one of the two main branches of modern physics. While general relativity provides a macro level perspective (space-time and gravity), quantum theory offers a micro level perspective that includes subatomic particles. Currently, quantum theory is used in a wide range of everyday applications, including lasers, optical fibres, digital cameras, transistors, superconductors, spectroscopy, and magnetic resonance scanners. More recently, the first book on quantum biology was published in 2014, which addressed the application of quantum mechanics and theoretical chemistry to biological objects and issues [3]. However, the integration of quantum principles in agriculture been negligible to date, although Lovel [4] had predicted in 2015 that the application of quantum principles in agricultural techniques could increase the efficiency of the raw materials used, improve animal welfare, and reduce adverse environmental consequences. Therefore, it desired that any positive development in this area will have a rapid and highly potential impact on enhancing productivity. The urgent challenge for research is to address a growing set of questions regarding the applications of quantum agricultural practices and the use of new technologies for measuring and analysing bio-photonic information and flows which are based on quantum principles and techniques. Functional analysis allows us to assess the relationship between crop productivity through agronomic surveys (height of the plant and length of the spike); and/or (i) qualitative improvement of yield; (ii) characterisation of production for homogeneous zones/territorial areas that can correspond to specific needs of the processing industry; and (iii) environmental factors; type of crop (quantum type; traditional).

## 2 Quantum agriculture: The state-of-the-art technology

Quantum physics has been discussed for a hundred years, but scientific research focused on this new discipline only in the last few decades, which subverts many of the cornerstones of traditional physics. From this perspective, it is no longer a matter of gaining access to new levels of knowledge and consciousness; rather, it is about discovering how far humans can progress in harmony with the laws of nature. Literature on the application of quantum physics to agriculture, reveals that research has been conducted on (i) the connection between quantum theory and agriculture particularly with regard to the analysis of wavelengths in agriculture [5], which is useful for evaluating the effects of sound in agriculture [6,7]; (ii) the connection between quantum physics and biology; and (iii) the link between quantum physics, crops, and livestock health [8,9]. Previous studies have examined the response of bacterial cells to sound [10], the relationship between infrared light and insect control [11], the effects of magnetised water on potted plants, and the effects of electromagnetic stimuli on cattle and fish [12]. Other studies have focused on the nutritional quality of food [13], general nutrition, soil health, and energy quality of food [14].

Notably some studies on crop and livestock productivity have evaluated the effects of sound and electromagnetic frequencies on wheat [15,16], other plants [17] and animals [18,19]. A study by De Souza et al*.* [20] on the effects of magnetic treatment on tomato seeds identified a wide range of physiological effects in response to magnetic fields, including positive effects on plant growth and development, enzymatic activity, protein synthesis, auxin content, water absorption, seed germination, fruit maturation, crop yield, and plant nutrient composition.

In this context, the quantum concept of networking is associated with the emerging field of research on intuitive agriculture, which incorporates the use of interspecies telepathic communication and/or meditative ability with cognitive ability and experience in making decisions in farm management. Long [21] first described interspecies telepathic communication in an academic context in 1919, and since then, numerous studies have provided evidence of this phenomenon, as reviewed by Erickson [22], McCraty et al. [23], and Kieft [24]. Many international organisations have been founded based on these principles and on the ability of human beings to integrate their interspecies communication into daily decisions; a few are mentioned here: Findhorn Foundation (Scotland), Tamera (Portugal), Perelandra Garden (USA), and Cooperative Biobalance (USA). In spite of growing empirical evidence demonstrating the effectiveness of intuitive agriculture in improving the production and resilience of agroecological systems, the scientific understanding of the mechanism, effects, and transfer of the skills required for intuitive agriculture is still in its infancy and requires substantial additional research.

In summary, the potential of the quantum approach in agriculture needs to be confirmed through scientific research, measurement, and evaluation. Studies funded by the European Union's Seventh Framework Program are currently underway in Europe on the use of ultrasound and music and its beneficial effects on food and products, bacterial disinfection through ultraviolet light, the use of bio-photonic techniques to test product quality throughout the food shelf life, treatments based on electromagnetism to improve animal health, and water treatment with electromagnetic frequencies.

This research landscape emphasises the need for multidisciplinary and transdisciplinary approaches to continue this quantum-type research orientation in agriculture, which is underexplored in the search of authentic, efficient, and effective alternatives to industrial agriculture.

With an aim to evaluate the potential of QA, the objectives of this study were two-fold. A quantum-based agronomic protocol proposed by Nastati [25,26] was first experimentally evaluated. Second, a thermal imaging measurement technique based on biophoton emissions was introduced by proposing a statistical analysis setting and interpreting the key statistical indicators.

## 3 Materials and methods

### 3.1 Research framework: new quantum based agronomic practice and biophoton measurement

**Fig. 1:**
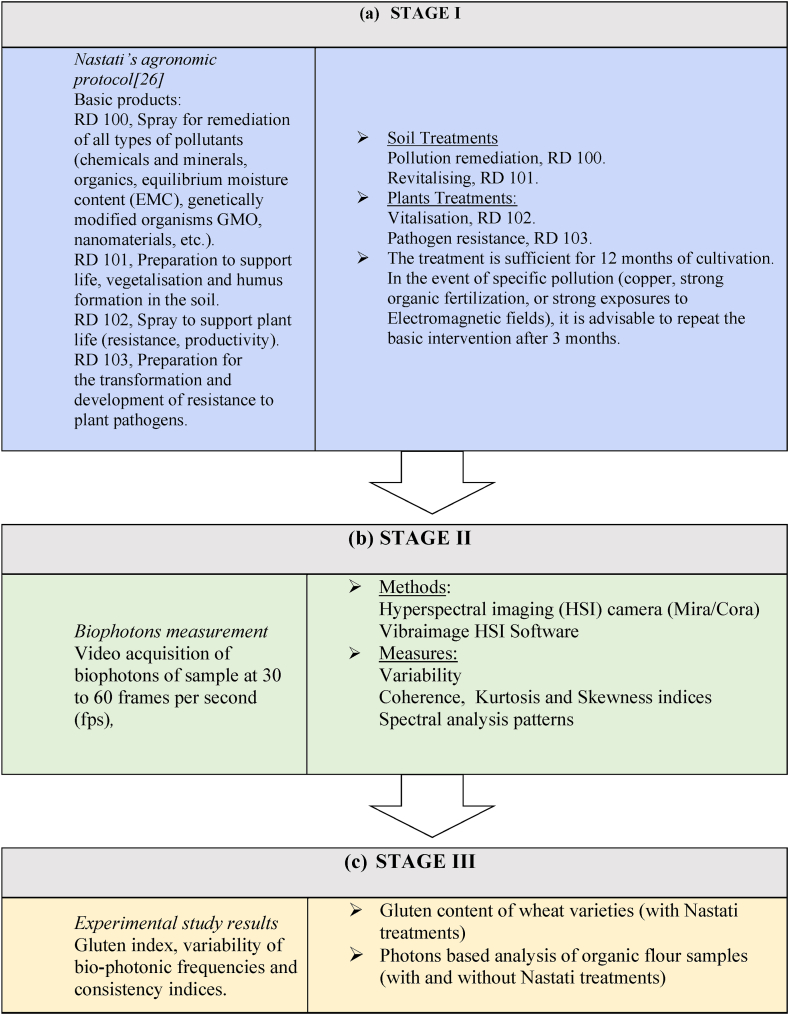
Flowchart of methodology for this research which includes (a) the quantum based agronomic protocol of Nastati [26] (Stage I), (b) the biophotons measurement and analysis (Stage II), (c) Experimental study results (Stage III).

In line with the first objective of this study, field experiments were conducted to demonstrate and evaluate a new quantum agronomic practise primarily aimed at reducing chemical inputs. The experimental protocol is based on QA vision and homoeopathic methodology derived from the theories and concepts of quantum metaphysics (Fig. 1). In line with QA vision, the second objective aimed to experimentally evaluate a multispectral analysis technique for the measurement phase and functional statistical analysis of wheat flour and/or processed products (such as pasta). A novel imaging technology was applied to analyse the functional and compositional information using each pixel of the image to determine the frequency, phase, and amplitude of biophoton emissions attributable to specific characteristics or the quality of wheat. Variable resonance camera technology measures the spectral distribution in real time using the Fourier transform by identifying the product's functional state. This measurement technique is non-destructive, permits obtaining reliable results rapidly, and also allows simultaneous determination of different components/dimensions using algorithms and statistical calibration processes, which can provide valuable information regarding the qualitative characteristics and dimensions of wheat flour. This research protocol was developed on the guiding principles that (i) it should be feasible to implement across different countries, environmental contexts, and over time; (ii) serve to establish links between the quality of the wheat and the biological characteristics of the product being examined in terms of biophoton emissions; and (iii) be capable of identifying the main measures (variance of rate of bio-photonic emissions, quantum coherence, entropy measures) and their interpretation using the nontrivial statistical properties of ultra-weak photon emission (biophotons). Fig. 1 depicts the basic steps of the proposed research protocol.

### 3.2 Stage I: Quantum based agronomic protocol

The proposed agronomic protocol is based on QA principles according to which there is a constant interaction between all the components of a system, and an imbalance in any component causes imbalance in the other “organs/components” of the system.

The farm is conceived as a unitary organism, which includes the soil, animals, and plants, as well as the farmer. Therefore, the bioenergy level of the various biological organisms that interact on farms can be rebalanced using homoeopathic remedies.

According to the quantum concept, there is a constant flow of information of an imponderable nature, the so-called “vibrations”, among all the components of the agricultural world. The farmer can benefit by scientifically analysing the dissonances and applying the right methods to restore the overall harmonic balance to promote the health of the various components of the farm and, consequently, its productivity.

In line with the principles of holistic medicine that a disease must be cured not by eliminating the symptoms but by acting on the entire human body to restore its equilibrium, biodynamic preparations serve as agents to balance the elements in biodynamic agriculture.

In this study we hypothesised the cultivation of wheat using methods that help the plant to connect as much as possible to its “species principle” by applying specific harmonising treatments directed at the seed, soil, and plant during its developmental stages through interventions that bring forces of silica and quality nitrogen so that the plant can “receive” adequate information to develop harmoniously and produce seeds compliant with the laws of nature and containing regenerated gluten.

Thus, we believe that we have taken the first steps to overcome the particle matter approach developed within the disciplines of chemistry and biology which focuses on the nature of the individual components of physical systems such as atoms, plant genes, soil-borne diseases, or water pollutants. In contrast, QA applied in this study draws on the theories and concepts of physics and quantum biology by adopting an approach based on the action of “waves” and the “information” of particles and their effects.

### 3.3 Stage II: Biophoton measurements

The proposed quantum statistical analysis is based on the results related to (i) the variability of bio-photonic frequencies (emissions/signals) and (ii) coherence measures obtained using MIRA/CORA, hyperspectral imaging software (HSI), and Vibraimage HSI. The bio-photonic emissions of the various wheat flour samples were used to measure the vitality levels (variability of the photonic frequency). In addition, the level of coherence associated with flour was derived from the waveforms of the variability of the photonic frequency, as well as the distribution relative to the power of the variability of the photonic frequencies (spectral density of the pattern), which indicates higher level of coherence of the product.

The bio-photonic and energy/vibrational properties of the samples were determined using a CCD/CMOS camera with a monochrome sensor with or without an HSI matrix and an artificial intelligence unit (microchip) called MIRA/CORA by Daniele Gullà’s HSI company [27]. This method is suitable for analysis of natural organic compounds or compounds with a predominant molecular H_2_O base.

This instrument uses a laser beam to scan organic materials. It acts as a vibrational liquid chromatograph in which optical detectors measure changes in the intensity of reflected light passing through a substance, and the pulses are transformed into an electrical signal transmitted to a computer that provides a variance graph very similar to a chromatogram (biophoton scattering).

To analyse the behaviour of photons in the environment (flow, directionality, reflectance, absorbance, and scattering), a basic method setup is required. Typically, it is operated in the dark or with extremely narrow-bandwidth filters to achieve a resolution of 0.2 nm.

Optoelectronic measurements were performed in a dedicated laboratory or in a clean room with controlled environmental conditions (stable light, temperature, and humidity and electromagnetic waves controlled within a Faraday protection structure) and a stable anti-vibration table and coherent homogeneous artificial lighting (laser light)). Multiple hundreds of frames were captured, magnified, and digitally analysed using a variety of proprietary algorithms (the system was patented in 2018 [28]). Light sources of different wavelengths ranging from near-UV to near infrared (NIR) wavelength can be used. Below is a schematic of the data summarisation process and the consequent interpretation following data visualisation on the monitor and Excel file storage. Multiple iterations of the test (at least three) produce “uninformed” and “informed” samples for analysis.

The measurements were performed in an enclosed space at a constant temperature to mitigate potential environmental variables. After collection and processing of the samples, several numerical parameters of these samples were acquired, and the average was computed on the image stack. In general, 5 to 6 primary parameters are analysed, but several other components can also be optionally registered using resident or external tools.

The principal parameters are:

• ADU count of photons by reflectance • Mean of T-scale variations • Variance • Standard deviation • Entropy • Max entropy • Kurtosis index • Vibrational spectrum (0.1 Hz–30 Hz) • Phase and wavelet analysis • Fractal size • Fractal spectrum • Harmonic analyser • Spectral correlation • Spectral angle • Spectral gradient Principal component analysis (PCA) • Spectral information divergence • Manhattan distance • Euclidean distance • Chebischev distance • Spectral gradient self-organizing map (SOM) • Vortex analysis.

The scattering biophoton reading device effectively captures the finest variations in light intensity, vibrational and spectral components, and can detect changes in the quantum characteristics of biological materials.

### 3.4 Stage III: Initial evidence and experiments

Since the autumn of 2016, the Eureka research institute and the Arc of Life association have collaborated with the New Land Cooperative of Codroipo (Ud) to produce the first tests for this study (Table 1). Experiments were conducted to improve gluten digestibility in two common varieties of wheat, soft wheat (Ludwig and spelt (Luni) in the New Land of Codroipo (Ud). The experimental fields were located in the Friulian Plain and employed certified organic farming techniques.

**Table 1:** Gluten analyses of wheat varieties.

Wheat Variety	Year	Gluten index
Farro dicocco Luni	2017	83.0
	2018	76.0
	2019	1.0
	2020	1.0
Ludwig soft wheat	2017	100.0
	2018	99.0
	2019	93.7
	2020	81.0

The protocol was developed by Nastati [26], the founder of the Eureka research institute and of the Arc of life association and provides products for interventions that have become an integral part of the patented eco-compatible Trinium method in agriculture patented by Nastati [26]. Interventions were administered on test cereals beginning with the seed treatment prior to sowing, followed by other treatments in the various stages of the vegetative cycle. The treatments were designed to aid plants in their cultivation cycle and ensure uptake of essential elements for wheat production and adequate flour yield for food in a harmonious way.

#### 3.4.1 The products used for the experiments

Using Nastati's protocol, the soil and plants underwent quantum regeneration [26]. Specifically, the homoeopathic techniques developed and illustrated by Hahnemann [29] in his “Organon of the art of healing” and Steiner [30] in his “Course of agriculture” were employed [31,32]. Based on the outcomes of human therapy using the formulations proposed by the “founding fathers” of homoeopathy, new formulations have been independently developed to make these products suitable for plants and animals. With lower evolutionary-energy level, plants and animals are less receptive to the messages carried by homoeopathic products, because they have different organic complexities, assimilation capacities, and metabolic rhythms.

The experiments in this study involved quantum treatment of both the soil and seeds of the selected test-plant varieties.

The soil treatments were carried out following a precise protocol in September, December, mid-February, mid-May, and before harvesting (July).

Products used for basic treatment of the soil to make it suitable for the modification of gluten in plants were.

1. RD 100: Pollution remediation product based on composted manure and appropriately enhanced plant derivatives.
2. RD 101: Product aimed at revitalising soil based on compost manure and appropriately enhanced plant and mineral derivatives.

Plants were treated for vitalisation and pathogen resistance with.

3. RD 102: A suitably enhanced product aimed at the plant vitalisation based on plant and mineral derivatives.
4. RD 103: A suitably enhanced product aimed at inducing resistance to pathogens based on iso-therapeutics obtained from the same pathogens.

## 4 Results

To date, the Luni spelt variety has been cultivated according to the Nastati protocol [26] for four consecutive years, in the New Land Cooperative of Codroipo (Ud). The results presented in Table 1, show that at the end of the first year (2017) the gluten index of Luni variety was 83, indicating very large gluten molecules. The gluten index was considerably reduced (76) in 2018, and in the third year of cultivation the index was very low with a value of 1, which was maintained in 2020 also.

However, the gluten modification response was slow in the Ludwig soft wheat grown according to the same Nastati protocol [26] for four consecutive years at the same location. As can be seen from Table 1, at the end of the first year the gluten index was 100 indicating very large gluten molecules. The value in the following year (99) was also comparable, which reduced to 93.7 in the third year of cultivation, and later in 2020 it was reduced to 81. All analyses were performed at the Laboratory of the Council for Research and Experimentation in Agriculture in Rome.

### 4.1 Statistical analysis and biophotons measurement

The first photon analysis experiment was conducted using samples of Ludwig variety organic flours (two quantum treated following Nastati protocol [26], one without Nastati treatment).

An HSI camera (Mira/Cora) and a laser/interferometry technique were used to map the variations in luminance and chrominance due to the micro vibrational activity contained therein. The objectives were to visually emphasise the image in the spatial and frequency domains (between 0.01 and 20 Hz) of the specific patterns and to statistically compare them to evaluate the significant differences. This technique involves acquiring a video of the sample for approximately 10–20 s at a sufficient sample rate of 30–60 frames per second (fps). Subsequently, the images acquired on the HSI matrix were analysed using a special software to verify the level of variance in brightness and chrominance. For this reason, polarised light (laser) was used to highlight the various biophoton-scattering characteristics reflected by the substances in a dark environment. The collected data were processed using a software. Several numerical parameters of the samples were acquired, and the average was computed on the image stack. Morphology and spatial heterogeneity are primarily analysed using micro-vibration-infrasonic after signal amplification by magnification, photonic scattering (Brillouin scattering), and using Fourier transform (FFT) of spectral components.

Finally, a differential comparison of the analyses was used to determine whether it is possible to quickly read the different electromagnetic responses of the specific and distinct patterns. The analyses were based on the results produced in terms of the variability of bio-photonic frequencies (emissions/signals) and consistency indices using MIRA/CORA and HSI Software [28].

**Fig. 2:**
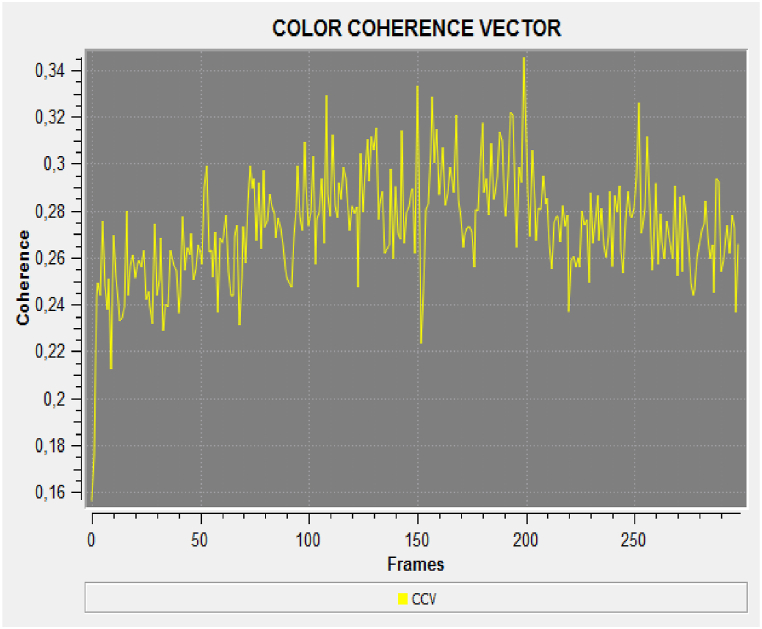
Coherence measurements of bio-photonic signals of “Bio standard, untreated Ludwig variety” (untreated Ludwig variety organic flour).

**Fig. 3:**
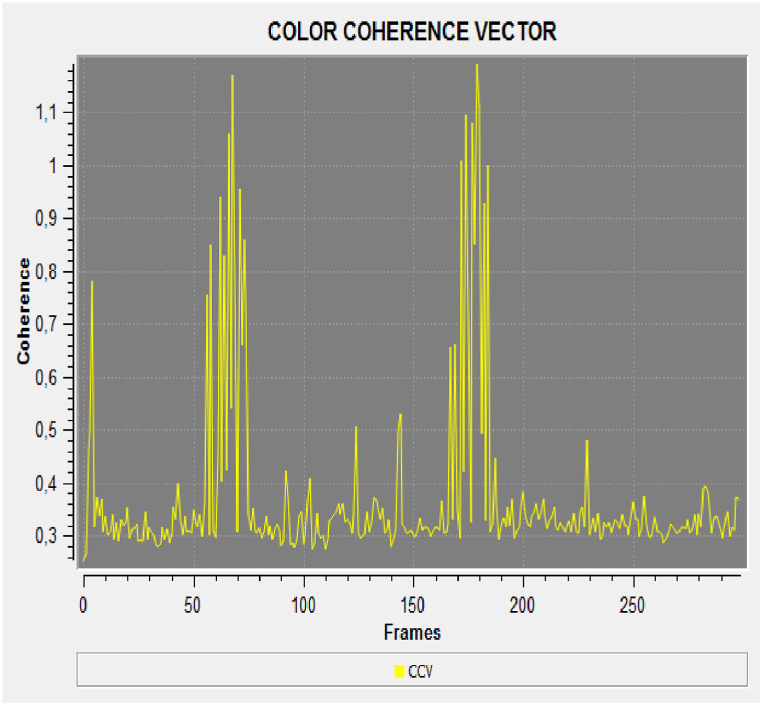
Coherence measurements of bio-photonic signals of “Bio standard, Ludwig variety with Nastati treatment” (treated Ludwig variety organic flour).

**Fig. 4:**
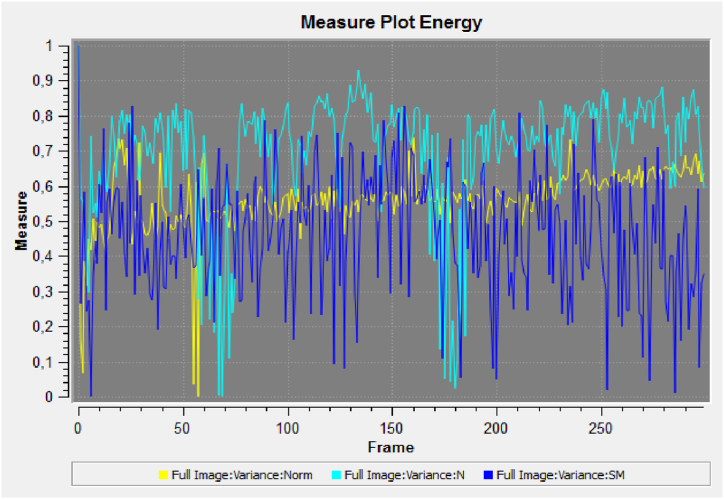
Global comparison of the vitality (variance) of the flour samples (Yellow, untreated Ludwig variety organic flour; light blue, treated Ludwig variety organic flour; dark blue, treated Ludwig variety organic flour).

**Fig. 5:**
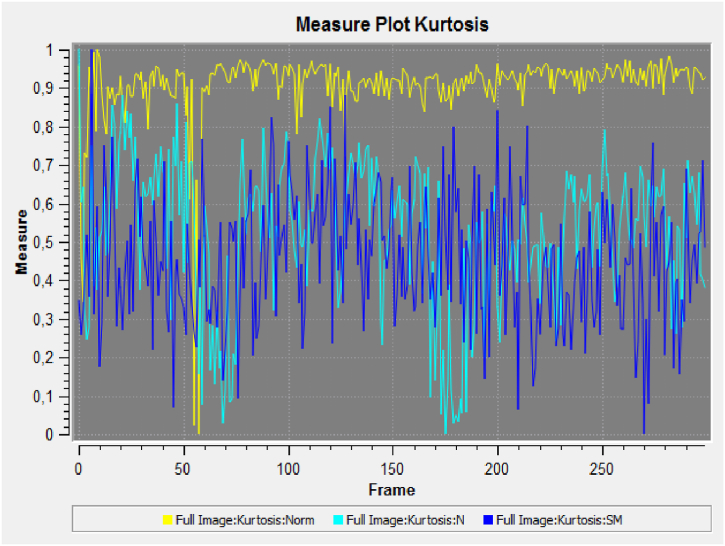
Kurtosis measurement of bio-photonic signals of the flour samples (Yellow, untreated Ludwig variety organic flour; light blue, treated Ludwig variety organic flour; dark blue, treated Ludwig variety organic flour).

**Fig. 6:**
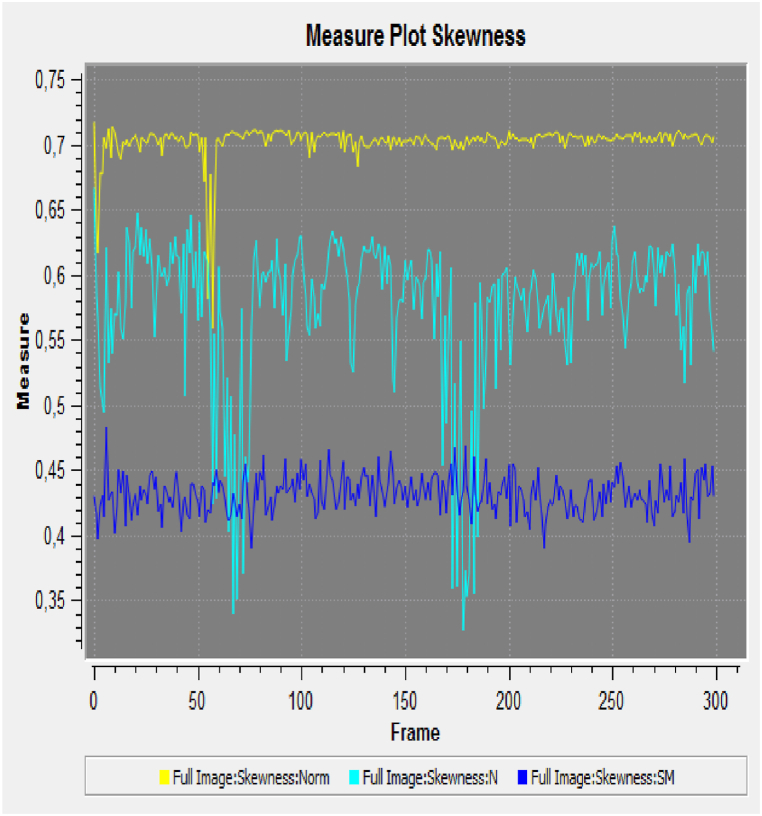
Skewness measures of bio-photonic signals of the of flour samples (Yellow, untreated Ludwig variety organic flour; light blue, treated Ludwig variety organic flour; dark blue, treated Ludwig variety organic flour).

Each flour sample showed a different response in terms of frequency and phase (amplitude) analyses. The coherence indicator was calculated in terms of luminescence variation. The results in Fig. 2, Fig. 3 indicate greater variations in terms of coherence of bio-photonic signals for all the products (the values for untreated Ludwig organic flour were between 0.22 and 0.34; and those for treated Ludwig variety Nastati samples were between 0.3 and 1.2). The skewness and kurtosis indices (Fig. 4, Fig. 5, Fig. 6) of the samples revealed differences, particularly between treated and untreated flour samples [33].

The results presented in Fig. 3 show that quantum treated flours have a greater vitality than that of untreated Ludwig variety flour (in yellow colour). A greater variance in the light blue and dark blue signals (Nastati treated Ludwig varieties) are indicative of greater vitality.

An additional analysis was also performed using the Vibraimage HSI Software which revealed significant differences in the variability of the photonic frequency. These results may be associated with the product vitality.

**Fig. 7:**
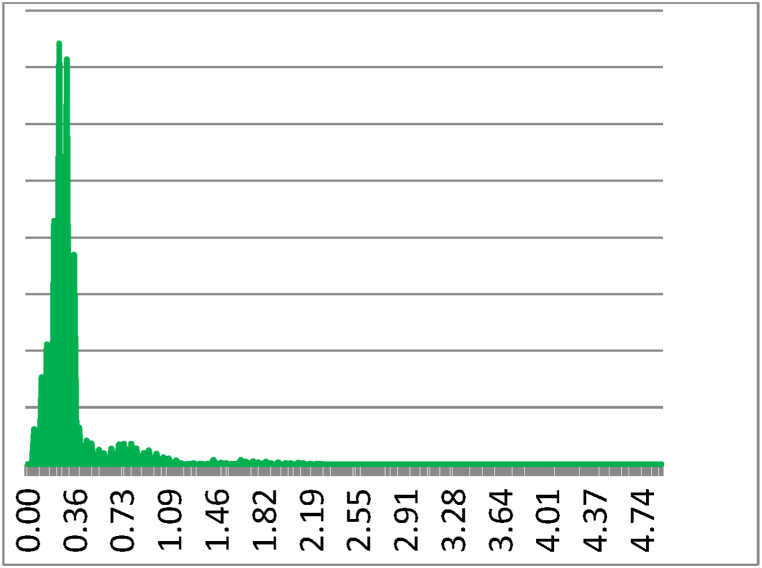
Frequency Histogram of Bio standard, untreated flour Ludwig variety.

**Fig. 8:**
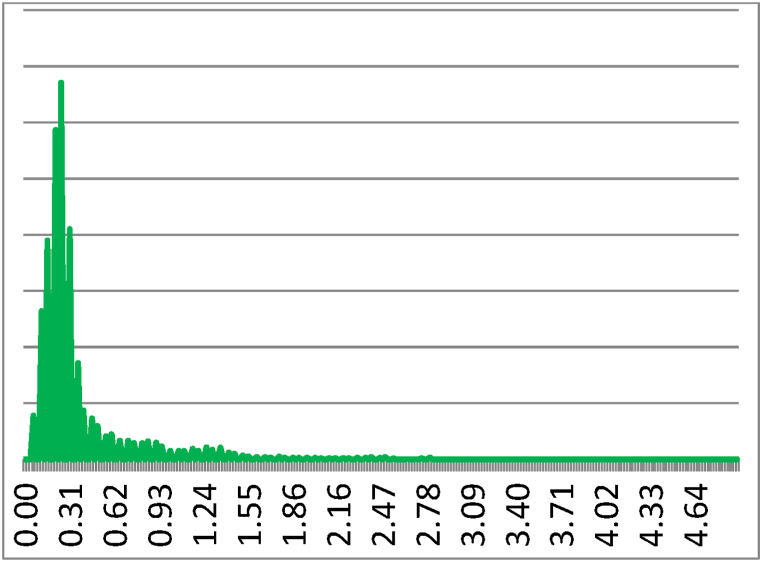
Frequency Histogram: Bio with Nastati_treatment, treated flour Ludwig variety.

The vitality values (variability of the photonic frequency) of Bio flour, Ludwig variety with Nastati treatment, registered much higher bio-photonic emissions, with the greatest relative variability equal to 1887.7 for the measured indicator. Whereas, for the untreated Bio standard flour (Ludwig variety), the indicator with the greatest relative variability was 32.864. This result is also confirmed by the wave shapes of the photonic frequency variability demonstrate a comparatively greater coherence of the photonic rhythm in the Nastati treated samples than in the untreated samples. Fig. 7, Fig. 8 indicate the extent of the variability in the biphotonic frequencies reported as the spectral densities of the pattern for flour, Ludwig variety, untreated and Nastati protocol treated samples, respectively.

Greater coherence is observed in samples of flour under Nastati treatment from the shape of the distribution (variability of the photonic frequency), which indicates an optimised state of energy consumption.

Our results provide an empirical basis for further extending this study to predict other important determinants of wheat quality, such as protein content, as a result of QA treatment using the proposed imaging technology devices.

Further experiments are required to replicate the results of this study under different environmental conditions. In addition, suitable comparative chemical analyses should improve the knowledge of the efficacy of the proposed agronomic practice in terms of toxicity and bioavailability.

## 5 Conclusions

1. This study demonstrates the application of quantum agriculture (QA) utilising optimal treatments (products and frequencies) by identifying the homoeopathic remedies appropriate for the crop and confirming any nutritional deficiencies, all in harmony with the environment.
2. This study introduces a rapid measurement device that can acquire signals with time and biophoton emissions consistently, which is useful for rapid and accurate measurement of the relationship between the quality of wheat and photon emission parameters.
3. In our view, the luminescence of biophotons, measured by biophoton emissions, is a quantum process involving a variety of functional parameters, and the biphoton luminescence distribution and their curve parameters can reveal information about the quality of wheat. The proposed technique makes it possible to correlate the quality of flour according to the adopted cultivation approach.
4. The results of this correlation analysis can help to define environmental friendly cultivation protocols capable of ensuring advantage to the farmers with respect to the crucial crop quality parameters such as gluten index, resistance to climate change and pathogens, and the use of limited water resources.
5. The novel research protocol based on quantum agronomic methodology and utilising bio-photonic measuring devices guarantees (i) a limited environmental impact, (ii) strengthening of key competencies, (iii) the transfer of technologies and knowledge, (iv) the capability to integrate quantum science and technologies into systems and services, and (v) the involvement of companies through a problem-solving perspective that encourages a multidisciplinary approach for holistic solutions to agricultural issues.
6. The preliminary experimental data seem to confirm the superior quality of wheat flour from the proposed QA based treatment. Within the quantum agriculture literature, the proposed practice is, to the best of our knowledge, a pioneer work.

## 6 Recommendations and future perspectives

There are still some limitations in this study which highlight future scope for research.

QA experiments under different environmental conditions can provide new insights related to the emerging challenges of water scarcity and potential soil desertification.

Comparative chemical analyses of the products should improve the knowledge of the efficacy of the proposed agronomic practice in terms of toxicity and bioavailability. A possible correlation between indicators based on biophoton measurements and those produced by standard agronomic, chemical, and nutritional analyses remains to be established.

The preliminary findings can be used to develop better quality of agricultural products and practices which are in harmony with nature. More specifically, two novel aspects have been highlighted in the study: the application of QA treatments of plant and soil for the cultivation of two wheat varieties and a novel quantitative analysis technique based on precise biophoton emission measurements of wheat flour obtained using the QA protocol. Such an integration of quantum principles in agriculture has thus far been negligible, although its application in agricultural techniques was intuited to have the potential to increase the efficiency of the raw materials used and animal welfare, while reducing adverse environmental consequences. Development in this area can lead to a highly potential and rapid impact for improved productivity.

Based on these results, it can be concluded that new quantum-based practices, including quantum homoeopathy, can play a crucial role in increasing plant productivity and food quality and ensure sustainable food production and environmental protection.

## Author contribution statement

R. Bernardini Papalia: Conceived and designed the experiments; Analysed and interpreted the data; Wrote the paper. D. Gullà: Conceived and designed the experiments; Performed the experiments; Analysed and interpreted the data; Wrote the paper. E. Nastati: Conceived and designed the experiments; Performed the experiments; Analysed and interpreted the data; Wrote the paper.

## Data availability statement

No data was used for the research described in the article.

## Additional information

No additional information is available for this paper.

## Declaration of competing interest

The authors declare that they have no known competing financial interests or personal relationships that could have appeared to influence the work reported in this paper.
